# Effect of Liquid Glass-Modified Lignin Waste on the Flammability Properties of Biopolyurethane Foam Composites

**DOI:** 10.3390/polym16020205

**Published:** 2024-01-10

**Authors:** Agnė Kairytė, Sylwia Makowska, Przemysław Rybiński, Krzysztof Strzelec, Arūnas Kremensas, Jurga Šeputytė-Jucikė, Saulius Vaitkus

**Affiliations:** 1 Laboratory of Thermal Insulating Materials and Acoustics, Institute of Building Materials, Faculty of Civil Engineering, Vilnius Gediminas Technical University, Linkmenų St. 28, 08217 Vilnius, Lithuania; arunas.kremensas@vilniustech.lt (A.K.); jurga.seputyte-jucike@vilniustech.lt (J.Š.-J.); saulius.vaitkus@vilniustech.lt (S.V.); 2 Institute of Polymer and Dye Technology, Faculty of Chemistry, Lodz University of Technology, Stefanowskiego 12/16, 90-924 Lodz, Poland; sylwia.czlonka@dokt.p.lodz.pl (S.M.); krzysztof.strzelec@p.lodz.pl (K.S.); 3 Institute of Chemistry, The Jan Kochanowski University, Żeromskiego 5, 25-369 Kielce, Poland; przemyslaw.rybinski@ujk.edu.pl

**Keywords:** biopolyurethane foam, lignin waste, liquid glass, coating, flammability, ignitability

## Abstract

Water-blown biopolyurethane (bioPUR) foams are flammable and emit toxic gases during combustion. Herein, a novel approach suggested by the current study is to use different amounts of lignin waste (LigW), which increases the thermal stability and delays the flame spread and sodium silicate (LG), which has foaming ability at high temperatures and acts as a protective layer during a fire. However, there have been no studies carried out to investigate the synergy between these two materials. Therefore, two different ratios, namely 1/1 and 1/2 of LigW/LG, were used to prepare bioPUR foam composites. The obtained bioPUR foam composites with a 1/2 ratio of LigW/LG exhibited inhibition of flame propagation during the ignitability test by 7 s, increased thermal stability at higher temperatures by 40 °C, reduced total smoke production by 17%, reduced carbon monoxide release by 22%, and increased compressive strength by a maximum of 123% and 36% and tensile strength by a maximum of 49% and 30% at 100 °C and 200 °C, respectively, compared to bioPUR foam composites with unmodified LigW. Additionally, thanks to the sufficient compatibility between the polymeric matrix and LigW/LG particles, bioPUR foam composites were characterised by unchanged or even improved physical and mechanical properties, as well as increased glass transition temperature by 16% compared to bioPUR foam composites with unmodified LigW particles, making them suitable for application as a thermal insulating layer in building envelopes.

## 1. Introduction

Currently, energy-efficient buildings have increased interest to global concerns about energy consumption. According to the literature [1,2], the building sector uses 40% of the total energy consumed for cooling and heating. Building materials with thermal insulation efficiently improve energy conservation. Therefore, these materials have been gaining more attention. One of the most extensively studied thermal insulating materials is polyurethane foam because of its advantages such as low apparent density, thermal conductivity, and superior mechanical performance [3,4,5]. However, the raw materials for polyurethane foams are obtained from petrochemical sources, which contribute to increased carbon dioxide emissions and negatively affect the environment [6]. Consequently, renewable resources to produce biopolyurethane (bioPUR) foams or bioPUR foam composites must be considered to ensure the balance between environmental impact and effectiveness of such thermal insulating materials for the building industry.

One of the few renewable raw materials that could be implemented to produce bioPUR foam composites is lignin waste (LigW), which is an abundant material given that it is a by-product of the large-scale paper and pulp industry. Based on common practice, LigW is used to supply power and heat. However, the burning of LigW only provides a short-term benefit, whereas using it as a renewable resource for bioPUR foam composites production assures long-term advantages [7,8]. Improving the value of LigW use and expanding the possible application areas of LigW are of great practical importance to reduce the negative impact on environmental pollution and increase the usage of renewable resources for natural polymers [9]. Although the hydroxyl groups in LigW particles can react with isocyanate to produce bioPUR foam composites, its direct use is still a great challenge due to aggregation, low solubility properties and reactivity problems in a polymeric matrix, and dimensional instabilities of the resulting products. Reactivity, aggregation, and solubility challenges can be overcome using the liquefaction technique [10,11], while dimensional instabilities of the resulting products can be eliminated by limiting the accessible hydroxyl groups by coating them with sodium silicate (liquid glass, LG) [12]. LG showed superior performance regarding the physical and mechanical properties of bioPUR foam composites with LG-coated LigW particles.

Several studies regarding the impacts of organic or organic-inorganic materials on the flame retardancy of various composites [13,14,15] have been published. Inorganic counterparts have become increasingly popular; however, LG has never been considered and adopted as a possible flame-retarding material applied as a coating material for fillers in polymer matrices. Its application as an intumescent flame retardant in geopolymers and its expansion properties were extensively studied by Chen et al. [16] who reported a 16 times expansion ratio when the LG and metakaolin weight ratio in the geopolymer was 9/1. Moreover, Kumar et al. [17] stated that LG forms a solid foam-like coating that protects the material against fire and can effectively inhibit the spread of the flame in wood coating materials. However, there is a lack of knowledge about the interactions of LG and the polymeric matrices during flame impact. Therefore, LG was selected not only to assure the dimensional stability of bioPUR foam composites with LigW particles but also to determine its effects on the flammability and thermal stability of such products. 

## 2. Materials and Methods

### 2.1. Materials

Two polyols (BioPolyol RD and PETOL 400-4G) were incorporated for the synthesis of bioPUR foam composites. The bio-based polyol (BioPolyol RD) was purchased from SIA PolyLabs, Riga, Latvia with a hydroxyl number of 350 KOH/g. The second polyol was a polyether polyol (PETOL 400-4G) purchased from Oltchim, Râmnicu Vâlcea, Romania with a hydroxyl number of 421 KOH/g. Lupranat M20S, a polymeric 4,4-diphenylmethane diisocyanate for use as a hardener, was supplied by BASF, Ludwigshafen, Germany. Distilled water was incorporated as an ecological blowing agent. Polycat 9 from Air Products and Chemicals, Inc., Allentown, PA, USA was added as a catalyst for the blowing and gelling reaction. ST-52, a silicone surfactant, was supplied by Shijiazhuang Chuanghong Technology Co., Ltd., Shijiazhuang, China. LigW was added as a filler in bioPUR foam compositions. Before coating with LG, it had 1 wt.% initial moisture content and a particle size of up to 1.25 mm. LigW was obtained from JSC Lignineko, Kėdainiai, Lithuania. LG was supplied by JSC Lerochemas (Na_2_O—29% and SiO_2_—29%), Klaipeda, Lithuania. After the treatment with LG, LigW/LG ratio 1/1 and LigW/LG ratio 1/2 particles had 0.3 wt.% and 0.4 wt.% moisture contents, respectively. The particle size of LigW/LG ratio 1/1 was up to 2.5 mm and for LigW/LG ratio 1/2 particles, it was up to 1.25 mm.

### 2.2. Preparation of bioPUR Foam Composites

LigW particles were coated with LG by spraying at 1/1 and 1/2 ratios, further mixed for 5 min at 1200 rpm, and dried for 24 h at 105 °C. The obtained LigW/LG particles were stored in sealed containers until the synthesis of bioPUR foam composites. 

First, the premix of two polyols was prepared by adding the scaled amounts of rapeseed oil-based polyol and sucrose-based polyol, distilled water, catalyst, and surfactant. Further, the final polyol premix was divided into four parts: one for control polyol premix, one for polyol premix with LigW particles, one for polyol premix with LigW/LG ratio 1/1 particles, and one for polyol premix with LigW/LG ratio 1/2 particles. Then, all polyol premixes were mixed with the intended amounts of LigW particles and LG-modified LigW particles. Lastly, the polyol premixes were thoroughly mixed with a scaled amount of isocyanate at 1800 rpm for 10 s. The prepared mixes were gently added into moulds and left to sit at 23(±2) °C temperature and 50(±5)% relative air humidity conditions to cool down.

### 2.3. Methods

A microstructural analysis of bioPUR foam composites and their char residues after the flammability test was completed using Helios NanoLab 650 (Oxford Instruments, Abingdon, UK). Before analysis, all samples were prepared by sputter coating their surfaces with a gold layer. The elemental composition of the char residues was determined using an X-ray spectrometer INCAEnergy (Oxford Instruments, Abingdon, UK) and an X-max detector.

The apparent density measurements were conducted according to the requirements of EN ISO 29470 [18] for samples which were cut out for compressive strength measurements.

The compressive strength was determined, and compressive stress-strain curves were obtained by EN ISO 29469 [19] using an H10KS Hounsfield universal testing machine (Tinius Olsen Ltd., Surrey, UK). Compressive stress at 10% deformation or compressive strength, whichever was greater, was determined at ambient temperature of 23 °C and at temperatures of 100 °C and 200 °C after conditioning samples at testing temperatures for 6 hours. Five samples of each bioPUR composition had a size of 50 mm × 50 mm × 50 mm.

A dynamic mechanical analysis (DMA) test was performed using an ARES rheometer (TA Instruments, New Castle, DE, USA). The temperature range was from 40 °C to 250 °C, with a frequency of 1 Hz, at a heating rate of 10 °C/min and a constant strain of 0.1%.

The test of long-term water absorption by total immersion was conducted according to EN ISO 16535 [20], method B requirements. Three samples of each composition were used. The size of the samples was 200 mm × 200 mm. Before the testing, all the samples were conditioned for 6 h at 23(±5) °C temperature. The water absorption test was carried out for 28 days in 20 °C water.

Differential thermogravimetric analysis (DTG) and thermogravimetric analysis (TGA) were implemented using an apparatus STA 449 F1 Jupiter Analyser (Netzsch Group, Selb, Germany). The bioPUR samples were tested at a heating rate of 10 °C/min in an argon atmosphere. The measurements were made in the 25–900 °C temperature range.

The heat release rate (HRR), total heat release (THR), total smoke production (TSR), carbon monoxide yield (COY), and carbon dioxide yield (CO_2_Y) were obtained according to ISO 5660-1 [21] using a cone calorimeter Cone 2a (Atlas Electric Devices Co., Chicago, IL, USA). Heat flow during the test was 35 kW/m^2^, with a testing time of 300 s.

The ignitability was conducted on three samples of each bioPUR composition with a size of 200 mm × 100 mm × 50 mm based on the method indicated in ISO 11925-2 [22]. All bioPUR samples were tested with an open flame which was directed at a 45°angle. The test was carried out for the standard 15 s. After the removal of the flame source, the bioPUR samples were left for 5 s to flame.

A limited oxygen index (LOI) test was conducted using NETZSCH TAURUS Co., Ltd., Weimar, Germany. The tip of the samples was ignited for 5 s with a propane–butane mixture burner. The LOI was calculated as the percentage of oxygen and nitrogen volume.

## 3. Results and Discussion

### 3.1. Physical and Thermo-Mechanical Properties of bioPUR Foam Composites

It is well known that polymeric foams undergo a degradation process at higher temperatures. Duarte et al. [23] studied the change of thermophysical properties such as thermal conductivity and specific heat at temperatures up to 1000 °C and proposed the numerical model allowing the prediction of the behaviour of polyurethane and polyethylene therapthalate foams-based sandwich panels under fire conditions. Our results showed that the variation in normalised thermal conductivity and specific heat increases with the rise in testing temperature. Therefore, the tests of temperature-dependent mechanical properties are of great importance.

The most relevant physical and mechanical properties of bioPUR foam composites are summarised in Table 1. The incorporation of LigW particles reduced the apparent density of the bioPUR foam composites. The maximum reduction, i.e., 21%, was observed for foam composites with 10 wt.% LigW particles. This can be explained by the available hydroxyl groups in LigW particles. The deficiency of isocyanate groups became significant, resulting in the loss of hard segment ordering and more unstable softer composite foams with reduced apparent density values [24,25]. However, Bartczak et al. [26] observed that the addition of 2.5–10 wt.% LigW particles resulted in an increased apparent density of flexible polyurethane foam. The differences occurred due to different cellular structures of produced foam, i.e., open-cell flexible vs. closed-cell rigid polyurethane foam. Opposite results were observed for LigW/LG ratio 1/1 and LigW/LG ratio 1/2 particles-modified bioPUR foam composites. The apparent density of foam composites with 2.5–5 wt.% LigW/LG ratio 1/1 particles remained unchanged compared to control bioPUR foam, although further addition increased the parameter by ~9%. Moreover, similar observations were made for bioPUR foam composites with LigW/LG ratio 1/2 particles. The difference in apparent density trend between bioPUR foam composites with LigW particles and bioPUR foam composites with LigW/LG ratios 1/1 and 1/2 particles could be attributed to the fact that the effect of available hydroxyl groups in LigW particles was eliminated by LG coating, thus significantly improving the dimensional stability of the final products [12].

**Figure 1:**
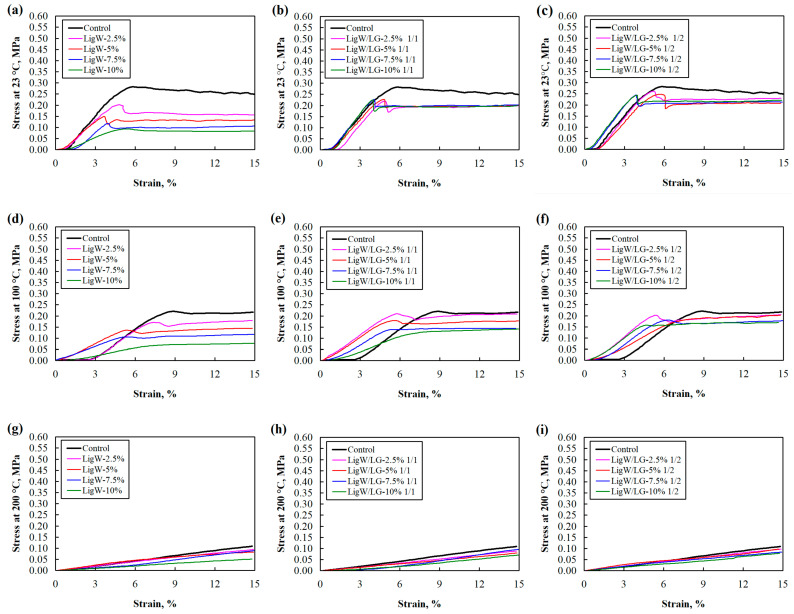
Stress-strain curves of bioPUR foam composites with LigW, LigW/LG ratio 1/1, and LigW/LG ratio 1/2 particles at (**a**–**c**) 23 °C, (**d**–**f**) 100 °C, and (**g**–**i**) 200 °C.

It is said that apparent density results mostly correlate with the mechanical properties of bioPUR foams and bioPUR foam composites [27,28], and the most suitable way to describe the non-linear materials such as polyurethanes is to analyse the stress-strain curves under different temperature conditions. Compressive strength results at ambient temperature were inconsistent except for LigW particles-modified bioPUR foam composites. The lower the apparent density that was obtained, the lower the values of compressive strength determined for bioPUR foam composites with LigW particles, which is also shown in Figure 1. 

Further, the compressive strength of LigW/LG ratios 1/1 and 1/2 particles-modified bioPUR foam composites decreased at higher values of apparent density. For instance, compared to 2.5 wt.% bioPUR foam composites, a deterioration of up to 4.3% and 7.7% was obtained for bioPUR foam composites with 10 wt.% LigW/LG ratio 1/1 particles and LigW/LG ratio 1/2 particles, respectively. It was assumed that the incorporation of either LigW or LigW/LG particles weakened the cellular structure of the resulting products due to solid particles located in cell walls and mostly the porous impurities of LigW particles. Additionally, our results showed that using the higher ratio of LigW and LG resulted in higher compressive values obtained at the same apparent density of the bioPUR foam composites, which was related to the cross-link formation between LG-coated LigW fillers and the bioPUR foam polymeric matrix [29].

According to Horak et al. [30], the decrease in the mechanical properties of polyurethane foams up to 90 °C is relatively small; therefore, further studies were carried out at higher temperatures such as 100 °C and 200 °C. For bioPUR foam composites with LigW particles, the maximum reduction in compressive and tensile strengths at 100 °C were 17% and 52%, respectively.

Moreover, it can be observed from Table 1 that the higher the addition of LigW particles, the lower the reduction in mechanical properties at 100 °C temperature. Different trends can be depicted for bioPUR foam composites with LigW/LG particles. When a greater amount of LG was used, there was a greater reduction in mechanical properties. For instance, the maximum reduction in compressive and tensile strengths of bioPUR foam composites with LigW/LG ratio 1/1 particles at 100 °C were 40% and 65%, respectively, while for bioPUR foam composites with LigW/LG ratio 1/2 particles, these values were 32% and 57%, respectively. Such changes may be associated with the thermal decomposition of LG, which has an onset degradation temperature slightly above 100 °C [31], thereby eliminating the proper contact zones between particles and polymeric matrix, as well as possibly reversing chemical aging of the polyurethane structure and macromolecular networks, as chain scission during thermal treatment becomes more important, thus leading bioPUR foam composites to lose their rigidity and resistance.

The most noticeable changes in mechanical properties can be noticed at 200 °C. Even the curves of stress-strain response in Figure 1g,h,i were more linear compared to the ones of control bioPUR foam and bioPUR foam composites at temperatures of 23 °C and 100 °C. This suggests that bioPUR foam composites fully lose their rigidity at 200 °C. In addition, the review by Wang et al. [32] on temperature impact on polyurethanes highlighted the most recent results that indicated the changing patterns of the compressive and tensile strengths of polyurethanes with different densities at different temperatures. Our results showed that the increase in service temperature also caused the loss of elastic modulus and strength of tested materials.

Furthermore, very promising results were obtained for long-term water absorption by total immersion. The lowest value was obtained for control foam. However, almost indistinguishable results were observed for LigW/LG ratio 1/1 and LigW/LG ratio 1/2-modified bioPUR foam composites, while LigW particles increased the parameter compared to bioPUR foams and all bioPUR foam composites. For example, 10 wt.% LigW particles increased water absorption by 1.3%, while the same amount of LigW/LG ratio 1/1 particles and LigW/LG ratio 1/2 particles by only 0.5% and 0.4%, respectively. The assumption can be made that LG effectively inhibited the penetration of water molecules into the LigW particles and their porous impurities, shown in Figure 1c, thus improving the hydrophobic nature of the filler used. Additionally, better water absorption values, as previously indicated, were characterised by higher closed-cell content of bioPUR foam composites with LigW/LG ratio 1/1 particles and LigW/LG ratio 1/2 particles. The improvement tendency was also observed in the study by Anwar et al. [33] with polyurethane foams reinforced with paper pulp.

**Figure 2:**
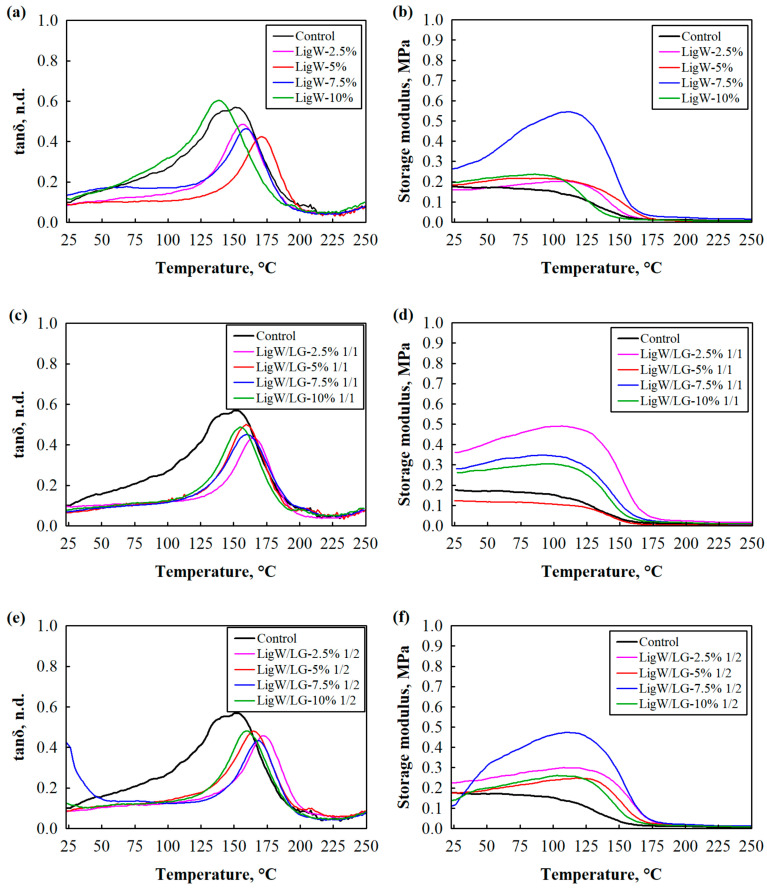
DMA curves of bioPUR foam composites with LigW, LigW/LG ratio 1/1, and LigW/LG ratio 1/2 particles: (**a**,**c**,**e**) tanδ and (**b**,**d**,**f**) storage modulus.

To measure the glass transition temperature and storage modulus, the DMA test was implemented. These results are shown in Figure 2 and summarised in Table 2. The addition of up to 7.5 wt.% unmodified LigW particles to bioPUR foam shifted the glass transition temperature to higher temperatures due to the constraints of polymer chains [34]. The maximum increase in T_g_ can be observed for 5 wt.% LigW-modified bioPUR foam composites (Figure 2a), while further addition of LigW particles reduced T_g_ to 140 °C, which is 5 °C lower compared to the control bioPUR foam due to the easier movement of the soft segments in the 10 wt.% LigW.

The same tendencies can be seen for the storage modulus of LigW-modified bioPUR foam composites (Figure 2b). The parameter increased with the addition of LigW particles up to 7.5 wt.%, i.e., from 0.16 MPa to 0.27 MPa at 23 °C and from 0.20 MPa to 0.53 MPa at 100 °C. However, 10 wt.% LigW particles reduced the storage modulus to 0.20 MPa and 0.23 MPa at 23 °C and 100 °C, respectively, which is higher compared to control bioPUR foam. Compared to control bioPUR foam, modification of bioPUR foam composites with LigW/LG ratio 1/1 particles (Figure 2c) increased T_g_ by a maximum of 22 °C at 2.5 wt.% loading, indicating better interaction between particles and the polymer matrix. Additionally, Figure 2d shows that the storage modulus of bioPUR foam composites at the same amount of LigW/LG ratio 1/1 particles increased by 0.18 MPa and 0.34 MPa at 23 °C and 100 °C, respectively, compared to the control bioPUR foam, while further loading of LigW/LG ratio 1/1 particles decreased the storage modulus. The highest T_g_ among all bioPUR foam composites was observed for the smallest amount of LigW/LG ratio 1/2 particles (Figure 2e). The glass transition temperature was shifted from 145 °C for control bioPUR foam to 175 °C for 2.5 wt.% LigW/LG ratio 1/2-modified bioPUR foam composites. This suggests that LG coating increased the ability of LigW particles to sufficiently interact with the polyurethane matrix. However, further loading of modified particles reduced the T_g_ but, in all cases, it remained higher than that of the control bioPUR foam. Surprisingly, the storage modulus value was rather low; for the 2.5–5 wt.% LigW/LG ratio 1/2 loading, it was higher only at 23 °C, but it increased at 100 °C for all LigW/LG ratio 1/2 particle loading compared to the control foam (Figure 2f).

### 3.2. Thermal Stability of bioPUR Foam Composites

**Figure 3:**
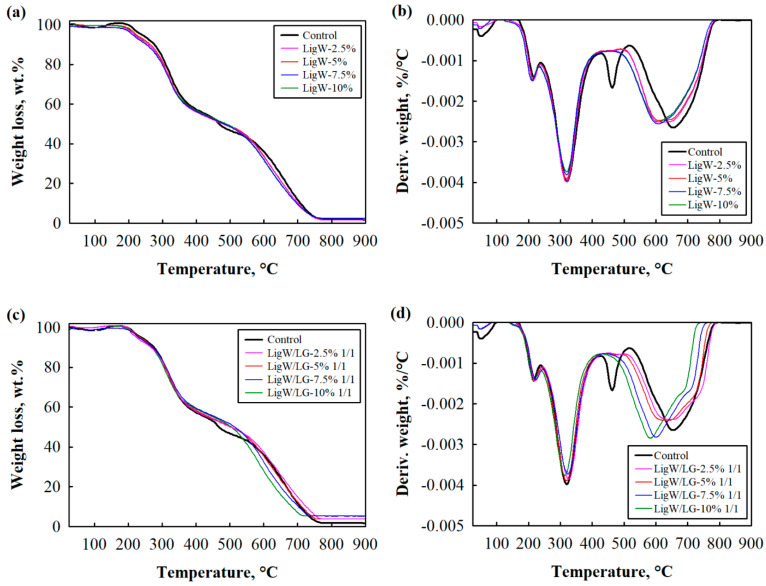

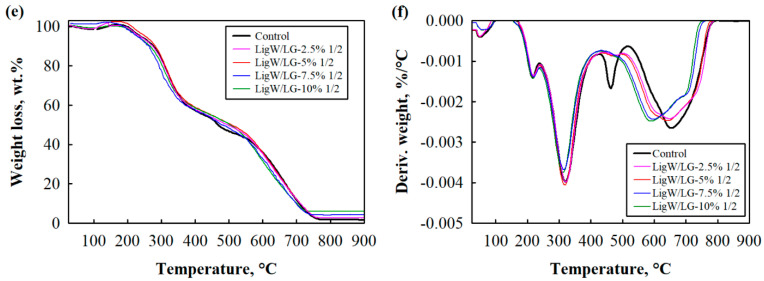
Thermal degradation patterns of bioPUR foam composites: (**a**) TGA of bioPUR with LigW, (**b**) DTG of bioPUR with LigW, (**c**) TGA of bioPUR with LigW/LG ratio 1/1 particles, (**d**) DTG of bioPUR with LigW/LG ratio 1/1 particles, (**e**) TGA of bioPUR with LigW/LG ratio 1/2 particles, and (**f**) DTG of bioPUR with LigW/LG ratio 1/2 particles.

The thermal resistance of bioPUR foam composites was evaluated by the TGA and DTG methods (Figure 3, Table 3). The shape of the curves remained unchanged with LigW or LG-modified LigW particle loading.

The weight gain at around 150–200 °C was observed due to the air buoyancy effect which gave the percentage greater than 100% in TGA curves. Further, the decomposition of urethane bonds and polyol chains started at ~200 °C [35,36] to form volatile products, while degradation of isocyanates and aromatic compounds occurred at higher temperatures [37]. At temperatures above 400 °C, previously generated fragments began to degrade with a 70% weight loss until 600 °C.

The TGA curves clearly showed that bioPUR foam composites with LigW/LG ratio 1/2 particles had the highest thermal resistance. The initial degradation temperature T_5%_ was 254 °C at 2.5 wt.% and 5 wt.% LigW/LG ratio 1/2, while the temperature T_50%_ was higher for all amounts of LigW/LG ratio 1/2 particles compared to the control bioPUR foam due to the barrier effect, which slows down the degradation of LigW/LG ratio 1/2 particles-modified composite foams. It was also observed that the addition of LigW and LigW/LG ratio 1/1 particles led to bioPUR foam composites with lower T_5%_ compared to control bioPUR foam. However, T_50%_ temperatures were higher regardless of the amount of LigW and LigW/LG ratio 1/1 particles, assuming that the thermal degradation shifted to higher temperatures for modified bioPUR foam composites. Our results are consistent with the observations of Ridho et al. [38] regarding the addition of LigW in polymeric systems. The analysis of the impact of LigW and LG-modified LigW particles on the thermal stability of bioPUR foam composites showed that the addition of either LigW/LG ratio 1/1 or LigW/LG ratio 1/2 particles increased T_50%_ by a minimum of 20 °C and 40 °C, respectively, compared to the control bioPUR foam. From these results, it can be concluded that LigW, LigW/LG ratio 1/1, or LigW/LG ratio 1/2 particles do not impact the main process of the thermal degradation of the bioPUR foam composites, but improve the thermal stability of the bioPUR foam composites. 

Most of the derivative curves of bioPUR foam composites (Figure 3b,d,f) were less intense and less resolved, indicating a slightly reduced degradation rate. However, they showed the same pattern. Similar findings were obtained by Luo et al. [28], who analysed the partial replacement of polyol with LigW particles in polyurethane foams. LigW has an aromatic structure and has previously been described as a potential flame retardant producing higher amounts of char upon heating, thus reducing the combustion heat [39,40].

This study showed that char yield increased with the addition of unmodified and LG-modified LigW particles. Table 3 shows that 10 wt.% LigW particles increased char yield up to ~2.5%, while the same amount of LigW/LG ratios 1/1 and 1/2 particles doubled the amount of residues at 900 °C, indicating a higher thermal stability of bioPUR foam composites with LG-modified LigW particles.

### 3.3. Ignitability and Flammability of bioPUR Foam Composites

**Figure 4:**
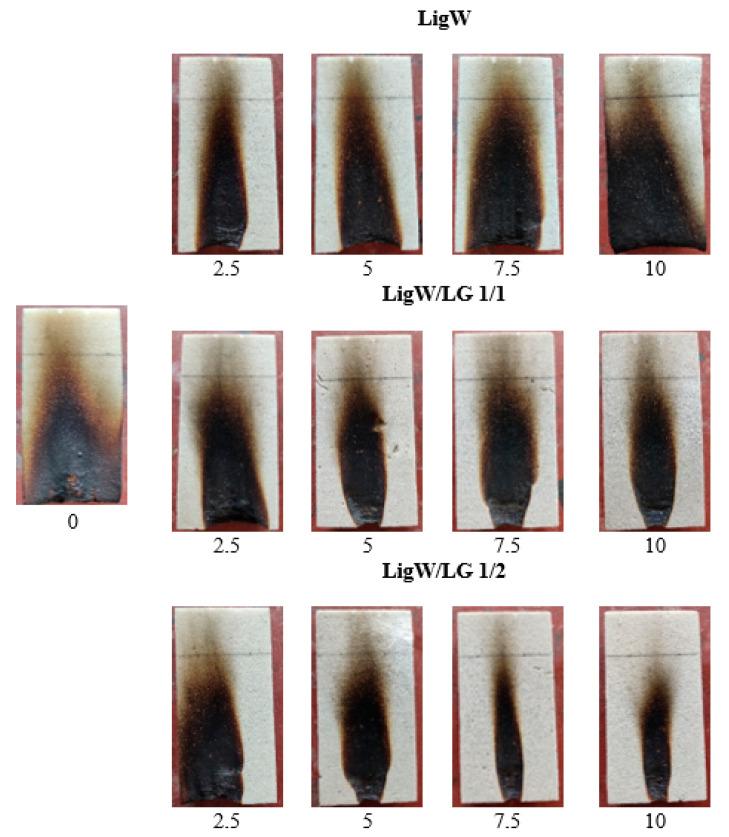
Images from the ignitability test on bioPUR foam and bioPUR foam composites modified with LigW, LigW/LG ratio 1/1, and LigW/LG ratio 1/2 particles.

Small-scale ignitability tests were implemented to determine the flame spread behaviour in unmodified and LG-modified LigW bioPUR foam composite systems. Figure 4 presents images of the burnt samples after the ignitability test. Additionally, Table 4 shows the main parameters obtained during the test.

Interestingly, it can be observed that the addition of unmodified LigW particles increased the flammability of bioPUR foam composites. The self-extinguishment was not achieved after the removal of the flame source and the height of the flame-damaged area increased. Therefore, the samples did not pass the test. However, the height of the damaged area was slightly lower compared to control bioPUR foam composites. These results agree with the study by Podkościelna et al. [41], who investigated the flammability of LigW polymer systems. They have concluded that the addition of natural unmodified LigW particles did not increase the rate of burning compared to control polymer systems. However, the time for the flame to reach 150 mm height was extended by approximately 5 s for all LigW-modified bioPUR foam composites.

A slight improvement was observed for the bioPUR foam composites with LigW/LG ratio 1/1 particles. At 10 wt.% particle loading, the height of the damaged area was reduced by 7 mm compared to 2.5 wt.% loading and by 23 mm compared to the control bioPUR foam. Self-extinguishment after 4 s and 5 s was also observed. Changing the ratio between LigW particles and LG to 1/2 showed that increasing the amount of LG resulted in an even better performance of bioPUR foam composites. 

The height of the damaged area was reduced by 21 mm at 10 wt.% loading compared to 2.5 wt.% loading and by 40 mm compared to the control bioPUR foam. Self-extinguishment after 3 s and 4 s was also observed.

**Figure 5:**
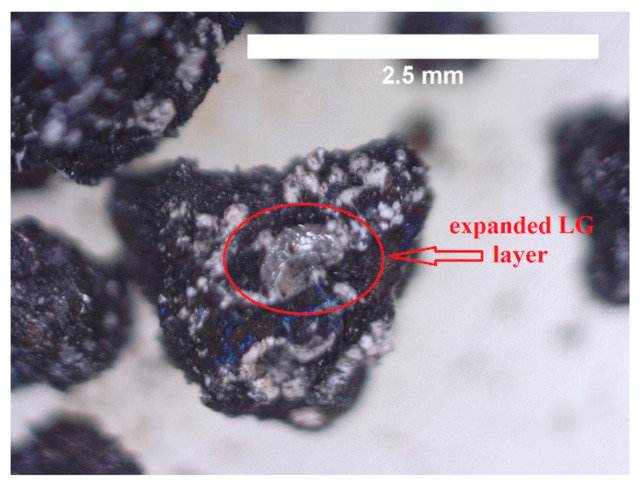
Expanded LG layer on LigW/LG ratio 1/2 particle after flame impact.

Moreover, the behaviour of LigW/LG ratio 1/1 and ratio 1/2 particles-modified bioPUR foam composites was different from that of LigW-modified composites. It can be noted from Figure 5 that, in the presence of a flame, the LG formed foam-like crystals that helped to provide an insulating barrier between the LG-modified LigW particles and the flame and thus slowed down the spread of flame [16]. The greater the amount of LG used to coat LigW particles, the better the flammability characteristics.

**Figure 6:**
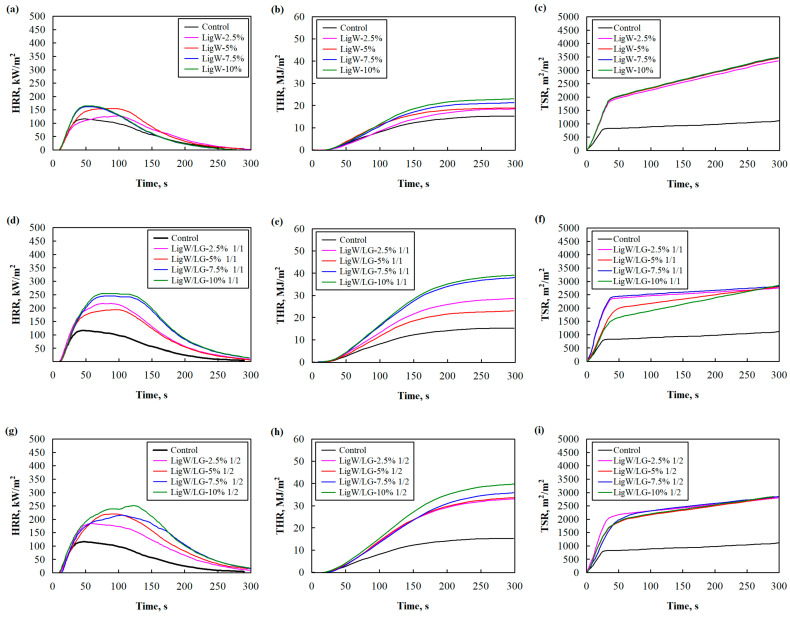
The main flammability curves of LigW, LigW/LG ratio 1/1, and LigW/LG ratio 1/2 particles of modified bioPUR foam composites: (**a**,**d**,**g**) HRR, (**b**,**e**,**h**) THR, and (**c**,**f**,**i**) TSR.

The cone calorimeter test for bioPUR foam composites was implemented as one of the best techniques to determine the behaviour of products under fire conditions and to obtain the relevant properties [42]. Consequently, the control bioPUR foam and LigW, LigW/LG ratio 1/1, and ratio 1/2 particles modified bioPUR foam composites were tested. Their HRR curves are presented in Figure 6a,d,g, THR curves are shown in Figure 6b,e,h, and TSR curves are shown in Figure 6c,f,i, while the relevant parameters are shown in Table 5.

The HRR, THR, and TSR curves demonstrated the burning behaviour of the bioPUR foam and bioPUR foam composites as a function of time. The addition of all LG-modified LigW particles had more intense HRR, THR, and TSR compared to control bioPUR foam. However, TSR curves indicated that the addition of LigW/LG particles reduced total smoke release compared to bioPUR foam composites with unmodified LigW particles. All the bioPUR foam composites showed higher values of pHRR (Table 5) compared to the control bioPUR foam. This could be related to the higher flammability of the bioPUR foam composites and the decomposition of LigW particles and the LG layer. Carretier et al. [43] also showed that the addition of higher amounts of LigW particles in polylactide and polyurethane elastomer matrices resulted in higher pHRR values. However, the increase in the amount of LG for the modification of LigW particles resulted in slightly lower pHRR (maximum reduction of 13%). From the pHRR results, it can be observed that the ratio of 1/1 is not sufficient for an effective LG layer to form on the LigW particle surface. The same trend is observed for the average values of THR.

Compared to the control bioPUR foam, the ignition time of LigW-modified bioPUR foam composites remained the same (Table 5). However, the ignition time of LigW/LG ratio 1/1 and 1/2 particles increased up to 5 s and 6 s, respectively, which was determined by the cellular structure and thermal conductivity of the resulting foam composites. Similar observations were made by Barczewski et al. [44], who studied the influence of expanded vermiculite on the fire behaviour of modified polyurethane foams.

The smoke emission of the control bioPUR and modified bioPUR foam composites was characterised by TSR as well as CO_2_ and CO emissions evaluation. LigW-modified bioPUR foam composites had TSR that was more than three times higher than the control bioPUR foam, while LigW/LG ratios 1/1 and 1/2 particles modified ones had two times higher TSR values compared to control bioPUR foam. LigW particles increased TSR, but LG helped to significantly reduce it. The same behaviour could be seen for CO_2_ and CO emissions for all compositions. Additionally, the CO/CO_2_ ratio shows the degree of complete combustion [45]. It is shown in Table 5 that the CO/CO_2_ ratio of bioPUR foam modified with LigW particles is higher compared to control bioPUR foam or LigW/LG ratios 1/1 and 1/2 particles-modified bioPUR foam composites. It can be assumed that LigW particles led to more incomplete combustion processes and greater toxicity of the emitted smoke.

LOI test is the method which is used to determine the flammability of polymeric materials. It shows the required oxygen concentration at which the material starts to burn [46]. All the obtained bioPUR foam composites independent of the filler type had an oxygen index above 21% (Table 5). It can be observed that the incorporation of the LG coating on LigW particles added an extra 3% for bioPUR foam composites with LigW/LG ratio 1/1 particles and approximately 4% for bioPUR foam composites with LigW/LG ratio 1/2 particles. The inorganic coating LG is a dehydrated glass, which, according to Wypych [47], has a LOI value of up to 32%, consequently increasing the flame-retardant effect.

To compare the results of this study, an additional literature review related to the use of biomass waste fillers, especially the ones that were modified, in polyurethane foams was conducted and presented in Table 6.

### 3.4. Microstructural Analysis of Char Residues From

**Figure 7:**
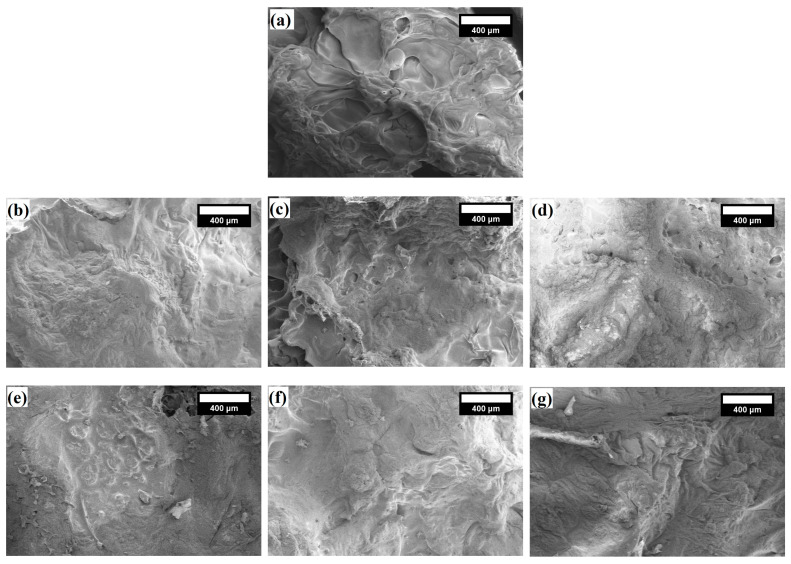
Microstructure of char residues of bioPUR foam composites after cone calorimetry (magnification ×100): (**a**) control, (**b**) LigW—5 wt.%, (**c**) LigW—10 wt.%, (**d**) 5 wt.% of LigW/LG ratio 1/1, (**e**) 10 wt.% of LigW/LG ratio 1/1, (**f**) 5 wt.% of LigW/LG ratio ½, and (**g**) 10 wt.% of LigW/LG ratio 1/2.

Analysis of the char residues can help to investigate the flame retardancy mechanism of LigW and LigW/LG ratios 1/1 and 1/2 particles-modified bioPUR foam composites because they theoretically could improve the flame retardance of polymers. Figure 7 shows SEM images of LigW, LigW/LG ratio 1/1, and LigW/LG ratio 1/2 particles-modified bioPUR foam composites after the cone calorimeter test.

As shown in Figure 7a, the char residue of control bioPUR foam displayed a loose surface, holes, and some cracks. These holes and cracks in the char layer contributed to insufficient isolation of heat and combustible gas. The char of bioPUR foam composites with LigW particles (Figure 7b,c) had a smoother surface; however, some irregularities and holes can be seen in the microstructure of char residues, which contributed to a slightly higher pHRR of such foam composites compared to the control bioPUR foam. The incorporation of LigW/LG ratio 1/1 particles (Figure 7d,e) resulted in a more irregular structure, which contributed to the highest pHRR values. Such a microstructure of char residues suggests that the char did not retard the heat and mass transfer efficiently during combustion [54]. As for char residues of LigW/LG ratio 1/2-modified bioPUR foam composites (Figure 7f,g), a more consistent, compact, and smooth surface of char residues can be observed. Compared to the char residues of LigW/LG ratio 1/1 particles, it is more effective to isolate the flammable gas and heat from the materials, thus leading to reduced pHRR. Additionally, the elemental composition of char residues after the cone calorimetry for all bioPUR foam composites was determined and the main elements are presented in Table 7.

Our results show that char residues of bioPUR foam composites have a higher content of sodium (up to 7.83%) and silica (1.82% to 5.67%), which is assigned to the LG coating. Due to its composition, it can act as a barrier for flame spread (Figure 5) by foaming its glassy film on the surface of LigW filler.

## 4. Conclusions

BioPUR foam composites were successfully synthesised with 2.5–10 wt.% unmodified LigW particles and different ratios of LigW/LG particles. The most significant results were obtained for bioPUR foam composites modified with LigW/LG ratio 1/2 particles. The incorporation of the latter particles resulted in long-term water absorption by total immersion ranging from 4.4% to 4.6%. Additionally, they improved the glass transition temperature by a maximum of 30 °C, storage modulus by 0.31 MPa at 100 °C with the 7.5 wt.% LigW/LG ratio 1/2 loading, and thermal stability at higher temperatures by a maximum of 40 °C, and increased the time for flame spread up to 13 s while reducing the height of flame damaged area from 138 mm to 98 mm. Moreover, such bioPUR foam composites had the highest LOI (up to 23.4%). According to our findings, the bioPUR foam composites can be successfully implemented as an in-situ sprayed or factory-made thermal insulating layer in building envelopes, conforming to the requirements of harmonised product standards. However, additional approaches should be taken into consideration for more significant improvement in HRR and TSR values. 

## Figures and Tables

**Table 1:** The summarised physical properties of bioPUR foam composites.

Lignin Waste, wt.%	Compressive Strength, kPa, at Different Temperatures			Tensile Strength, kPa, at Different Temperatures			Apparent Density ^1^, kg/m^3^	Water Absorption, vol.%
	23 °C ^1^	100 °C	200 °C	23 °C ^1^	100 °C	200 °C		
0	282 ± 5	220 ± 7	75 ± 5	231 ± 4	175 ± 5	104 ± 6	42 ± 2	4.2 ± 0.2
bioPUR foam with LigW								
2.5	206 ± 7	172 ± 6	66 ± 6	190 ± 5	133 ± 6	107 ± 5	38 ± 3	5.0 ± 0.4
5	150 ± 6	142 ± 8	66 ± 5	207 ± 6	144 ± 5	78 ± 5	36 ± 2	5.0 ± 0.3
7.5	113 ± 8	107 ± 5	56 ± 5	243 ± 3	137 ± 4	78 ± 6	35 ± 2	5.3 ± 0.3
10	95 ± 4	75 ± 6	36 ± 6	243 ± 4	117 ± 5	54 ± 4	33 ± 2	5.5 ± 0.3
bioPUR foam with LigW/LG ratio 1/1								
2.5	235 ± 9	210 ± 8	60 ± 4	208 ± 5	137 ± 4	77 ± 4	42 ± 3	4.3 ± 0.3
5	230 ± 8	179 ± 8	52 ± 7	282 ± 6	132 ± 5	72 ± 6	42 ± 3	4.5 ± 0.2
7.5	228 ± 5	146 ± 6	54 ± 6	284 ± 5	117 ± 6	63 ± 3	45 ± 2	4.5 ± 0.2
10	225 ± 6	134 ± 7	42 ± 5	286 ± 7	101 ± 3	50 ± 3	46 ± 2	4.7 ± 0.3
bioPUR foam with LigW/LG ratio 1/2								
2.5	260 ± 7	202 ± 6	67 ± 6	298 ± 6	198 ± 5	138 ± 6	43 ± 4	4.4 ± 0.2
5	247 ± 7	196 ± 7	65 ± 5	302 ± 6	167 ± 4	107 ± 4	44 ± 2	4.3 ± 0.2
7.5	241 ± 4	170 ± 5	59 ± 5	290 ± 4	141 ± 4	68 ± 5	46 ± 3	4.5 ± 0.3
10	244 ± 9	167 ± 8	49 ± 7	300 ± 5	130 ± 6	63 ± 3	46 ± 2	4.6 ± 0.2

^1^ NOTE: The designated properties were previously published in Kairytė et al. [12].

**Table 2:** Summarised results of the DMA test.

Lignin Waste, wt.%	T_g_, °C	Storage Modulus, MPa	
		at 23 °C	at 100 °C
0	145	0.18	0.15
bioPUR foam with LigW			
2.5	158	0.16	0.20
5	172	0.18	0.21
7.5	161	0.27	0.53
10	140	0.20	0.23
bioPUR foam with LigW/LG ratio 1/1			
2.5	167	0.36	0.49
5	161	0.12	0.11
7.5	161	0.28	0.34
10	157	0.26	0.30
bioPUR foam with LigW/LG ratio 1/2			
2.5	175	0.23	0.29
5	167	0.18	0.24
7.5	169	0.11	0.46
10	162	0.14	0.26

**Table 3:** Thermal degradation results of bioPUR foam composites.

Lignin Waste, wt.%	T_5wt.%_, °C	T_50wt.%_, °C	T_max_ Stages, °C				Char Yield at 900 °C, wt.%
			1st	2nd	3rd	4th	
0	240	468	221	324	464	660	1.68
bioPUR foam with LigW							
2.5	216	478	212	324	-	650	1.60
5	221	487	216	324	-	627	1.62
7.5	216	482	212	319	-	615	2.49
10	216	487	216	319	-	608	2.51
bioPUR foam with LigW/LG ratio 1/1							
2.5	235	502	216	326	-	663	3.68
5	235	505	221	324	-	650	3.76
7.5	232	506	228	319	-	598	5.24
10	230	506	221	314	-	585	5.29
bioPUR foam with LigW/LG ratio 1/2							
2.5	254	511	228	322	-	647	2.45
5	254	506	227	319	-	650	2.68
7.5	228	503	218	317	-	623	4.30
10	227	502	212	314	-	596	5.98

**Table 4:** Data from the ignitability test on bioPUR foam composites.

Lignin Waste, wt.%	Time for Flame to Reach 150 mm Height, s	Height of Flame Damaged Area, mm	Time of Self-Extinguishment after Flame Source Removal, s
0	6 ± 2	138 ± 4	-
bioPUR foam with LigW			
2.5	11 ± 2	109 ± 2	-
5	10 ± 2	125 ± 3	-
7.5	10 ± 3	130 ± 5	-
10	9 ± 2	140 ± 3	-
bioPUR foam with LigW/LG ratio 1/1			
2.5	10 ± 2	122 ± 2	5 ± 2
5	10 ± 3	120 ± 3	5 ± 2
7.5	10 ± 2	117 ± 2	4 ± 2
10	14 ± 3	115 ± 4	4 ± 2
bioPUR foam with LigW/LG ratio 1/2			
2.5	11 ± 2	119 ± 2	4 ± 2
5	12 ± 2	116 ± 3	4 ± 2
7.5	11 ± 2	109 ± 4	3 ± 2
10	13 ± 3	98 ± 2	3 ± 2

**Table 5:** Results from cone calorimeter test for bioPUR foam composites.

Lignin Waste, wt.%	pHRR, kW/m^2^	THR, MJ/m^2^	TSR, m^2^/m^2^	CO_2_Y, kg/kg	COY, kg/kg	COY/CO_2_Y, n. d.	Ignition Time, s	LOI, %
0	82	13.6	1141	3.67	0.18	0.05	3	19.8
bioPUR foam with LigW								
2.5	89	17.1	3422	3.88	0.32	0.08	3	21.0
5	102	17.7	3462	4.01	0.28	0.07	3	21.4
7.5	116	18.3	3479	4.12	0.34	0.08	3	21.6
10	125	19.7	3488	4.22	0.36	0.09	3	21.6
bioPUR foam with LigW/LG ratio 1/1								
2.5	159	25.8	2745	4.14	0.24	0.06	2	22.4
5	133	25.7	2789	4.26	0.29	0.07	3	22.4
7.5	184	31.7	2820	4.39	0.28	0.06	5	22.6
10	190	32.7	2843	4.53	0.32	0.07	5	22.8
bioPUR foam with LigW/LG ratio 1/2								
2.5	150	29.5	2815	4.38	0.22	0.05	3	23.1
5	152	29.9	2832	4.54	0.25	0.06	3	23.3
7.5	159	32.3	2855	4.69	0.28	0.06	6	23.4
10	173	36.8	2874	4.72	0.28	0.06	6	23.4

**Table 6:** Relevant findings for the flammability and thermal stability of polyurethane foam composites.

Waste Filler	Waste Filler Amount, wt.%	Results	Reference
Sunflower husk	5–15	Thermal stability at higher temperatures decreased by 4–13 °C, time to ignition (TTI) increased by 5 s, pHRR decreased by a maximum of 6 kW/m^2^, and LOI did not change significantly	[27]
Rice husk	5–15	Thermal stability at higher temperatures increased by 5–21 °C, TTI increased by a maximum of 10 s, pHRR decreased by a maximum of 5 kW/m^2^, and LOI did not change significantly	[48]
Buckwheat husk	5–15	Thermal stability at higher temperatures increased by 2–5 °C, TTI increased by a maximum of 8 s, pHRR decreased by a maximum of 12 kW/m^2^, and LOI did not change significantly	[49]
Rice husk with aluminium hydroxide	5	LOI increased by 4%, thermal stability at higher temperatures increased by 173 °C, and pHRR decreased by 73 kW/m^2^	[48]
Rice husk with aluminium diethylphosphinate	5	LOI increased by 3%, thermal stability at higher temperatures increased by 132 °C, and pHRR increased by 3 kW/m^2^	[48]
POSS-impregnated sugar beet pulp	1–5	Thermal stability at higher temperatures increased by a maximum of 11 °C, pHRR decreased by a maximum of 105 kW/m^2^, TSR decreased by a maximum of 400 m^2^/m^2^, LOI increased by 1%, and CO_2_Y and COY decreased by a maximum of 0.05 kg/kg	[49]
Walnut shells	5	Thermal stability at higher temperatures increased by 5 °C	[50]
Hazelnut shells	4–25	Thermal stability at higher temperatures decreased by 9 °C	[51]
Walnut shells treated with perlite, montmorillonite, and halloysite	2	Thermal stability at higher temperatures increased by 15 °C for walnut shells/perlite, 7 °C for walnut shells/montmorillonite, and 19 °C for walnut shells/halloysite. TTI increased by 4 s for walnut shells/perlite, 3 s for walnut shells/montmorillonite, and 4 s for walnut shells/halloysite. pHRR decreased by 29 kW/m^2^ for walnut shells/perlite, 32 kW/m^2^ for walnut shells/montmorillonite, and 31 kW/m^2^ for walnut shells/halloysite. TSR decreased by 187 m^2^/m^2^ for walnut shells/perlite, 242 m^2^/m^2^ for walnut shells/montmorillonite, and 356 m^2^/m^2^ for walnut shells/halloysite. COY did not change significantly. CO_2_Y decreased by 0.08 kg/kg for walnut shells/perlite and walnut shells/montmorillonite, and by 0.09 kg/kg for walnut shells/halloysite	[52]
Organosolv and kraft lignin from various sources	20	Thermal stability at higher temperatures increased by a maximum of 25 °C	[53]

**Table 7:** Relevant elemental composition of char residues of bioPUR foam composites after the cone calorimeter test.

Lignin Amount, wt.%	The Main Elements, %				
	C	N	O	Na	Si
bioPUR with LigW					
5	80.30	6.01	11.63	0.4	1.82
10	84.27	4.58	9.23	0	1.92
bioPUR with LigW/LG ratio 1/1					
5	80.65	7.04	9.21	0.29	2.81
10	78.71	5.12	13.12	0.04	3.01
bioPUR with LigW/LG ratio 1/2					
5	63.29	8.75	17.59	6.90	3.47
10	60.99	6.24	19.27	7.83	5.67

## Data Availability

Data are contained within the article.
